# Analyzing a phenological anomaly in *Yucca* of the southwestern United States

**DOI:** 10.1038/s41598-021-00265-y

**Published:** 2021-10-21

**Authors:** Laura Brenskelle, Vijay Barve, Lucas C. Majure, Rob P. Guralnick, Daijiang Li

**Affiliations:** 1 Florida Museum of Natural History, University of Florida, Gainesville, FL USA; 2 Department of Biology, University of Florida, Gainesville, FL USA; 3 Department of Biological Sciences, Louisiana State University, Baton Rouge, LA USA; 4 Center for Computation and Technology, Louisiana State University, Baton Rouge, LA USA

**Keywords:** Ecological modelling, Plant ecology

## Abstract

*Yucca* in the American desert Southwest typically flowers in early spring, but a well-documented anomalous bloom event occurred during an unusually cold and wet late fall and early winter 2018–2019. We used community science photographs to generate flowering presence and absence data. We fit phenoclimatic models to determine which climate variables are explanatory for normal flowering, and then we tested if the same conditions that drive normal blooming also drove the anomalous blooming event. Flowering for *Yucca brevifolia* (Joshua tree) and *Yucca schidigera* (Mojave yucca) is driven by complex, nonlinear interactions between daylength, temperature, and precipitation. To our surprise, early-season flowering odds are highest in colder and drier conditions, especially for Joshua trees, but increase with precipitation late-season. However, the models used to fit normal blooming overpredicted the number of anomalous blooms compared to what was actually observed. Thus, predicting anomalous flowering events remains a challenge for quantitative phenological models. Because our model overpredicted the number of anomalous blooms, there are likely other factors, such as biotic interactions or other seasonal factors, which may be especially important in controlling what is presumed to be rare, out-of-season flowering in desert-adapted *Yucca*.

## Introduction

In the winter of 2018–2019, park rangers and visitors to Joshua Tree National Park were surprised to see Joshua tree (*Yucca brevifolia* Engelm.) and Mojave yucca (*Yucca schidigera* Roezl ex Ortgies) in bloom. These two species normally bloom in spring from March to May^1,2^, and this anomalous bloom was unexpected and novel—so much so that this blooming event made local news^3^. While these reports anecdotally pointed to an unusually wet and cold fall as the reason for these *Yucca* being “fooled” into blooming, little is known about which environmental cues, and how cues may interact, to drive onset of blooming in *Yucca* or other arid-adapted plants.

Plant phenology in desert environments has been less comprehensively studied compared with temperate forests or even grasslands and prairies^4^. As might be expected, plants in arid regions have been shown to be particularly reliant on winter precipitation for plant growth during spring^5–8^. More formal phenological modeling of desert-adapted woody perennial species is surprisingly limited. Nevertheless, previous work has pointed to the potential for complex context-dependence between photoperiod, temperature, and precipitation. For example, Renzi et al. hypothesized that the onset of flowering in saguaro cacti (*Carnegiea gigantea* (Engelm.) Britton & Rose) may be triggered by rainfall followed by favorable temperature conditions.

Specifically within *Yucca*, previous work by multiple authors has focused on local or regional examination of desert *Yucca* phenology, but without a strong statistical framework for examining drivers of flowering onset^6,10–14^. *Yucca elata* (Engelm.) and *Y. baccata* (Torr.) have been shown to have increased reproductive output with increased winter and spring precipitation, with the slowest growth in early to mid-summer during what is often the driest season^10,11^. However, these studies did not focus on how environmental cues determine bloom timing. Ackerman (1980) tracked phenological timing of buds, open flowers and fruits in populations of *Y. schidigera* and *Y. brevifolia*, along with many other species, in southern Nevada over two years in relation to climate but did not develop formal models. He suggested that flowering in *Y. schidigera* may be driven by photoperiod. In *Y. brevifolia*, it has been shown that increasing temperatures may increase flowering density and fruit mass, but may also decrease the possibility of successful establishment of new plants^13^.

Formal modeling approaches that can test the relative importance of temperature, precipitation, and photoperiod as drivers of timing of *Yucca* phenology are critically missing. Such models are particularly important, because species of *Yucca* have highly specific, co-evolved obligate pollinators and herbivores—the yucca moths in the family Prodoxidae—and thus, their phenologies must synchronize in order to set fruit^15^. Given the constraints of arid environments and on *Yucca* in particular with obligate moth pollinators, our hypothesis was that there are tight environmental controls on blooming. However, previous evidence for synchrony between moth pollinators and *Yucca* flowering has been mixed, with strong synchrony demonstrated in some^16^ but not all *Yucca*^17^. Determining environmental factors affecting *Yucca* blooming is a key step towards understanding the possibility for phenological mismatches with their obligate pollinators. Models are also essential for assessing whether anomalous climate events are responsible for atypical timing of *Yucca* flowering, such as that which occurred in 2018. If anomalous blooms become more common due to changing environmental conditions or more extreme climate variation, this could have negative consequences for *Yucca*, given its specialized pollination system.

The biggest bottleneck for creating predictive models of phenology is availability of data across the range of a species and environmental gradients, but new data resources hold great promise for overcoming the data gaps. Most knowledge of desert plant phenology is derived from population studies at a limited number of sites^6,9,14,18–21^. While these studies are of high value and often provide detailed local phenological information, they do not often provide enough variation to capture the full range of conditions needed to test environmental drivers. Fortunately, availability of community science photographs of plants is growing exponentially and provides key phenological trait information at broad scales and with increasing data density. For example, as of December 2020, the community science platform iNaturalist^22^ has provisioned over 35,000 research-grade records of *Yucca* species. *Yucca* are also often photographed with a whole plant in view, providing crucially needed information about flower absence.

Here we scored the presence and absence of closed and open flowers of *Y. schidigera* and *Y. brevifolia*—the two species with documented anomalous blooms in fall-winter 2018—using best practice approaches for community science photographs described in 1. We linked these georeferenced and time-stamped records with key climate covariates including growing degree days (GDDs), precipitation and photoperiod. We considered two different GDD and precipitation accumulation periods, 30 and 120 previous days (before the observation date of an open flower), to determine if there are lags in response given the presumed importance of fall and winter rains on spring phenologies. We utilized a general linear modeling framework that included attention to model calibration and validation and that is focused on determining the probability of open flower over the year and in relation to climatic factors. This framework provides the means to address the following questions:

1. What are the key climatic drivers for the flowering phenology of *Y. brevifolia* and *Y. schidigera*? Do interactions between drivers lead to non-linear responses?
2. Can phenology models that are known to be performant in predicting normal flowering be applied to accurately predict the observed anomalous blooms in the fall and winter of 2018–2019?
3. Are models that take into account longer periods of heat or precipitation more accurate than those that use shorter-term accumulations?

## Results

### Summary of spatial distribution of records and shape of seasonal phenology curves

**Figure 1:**
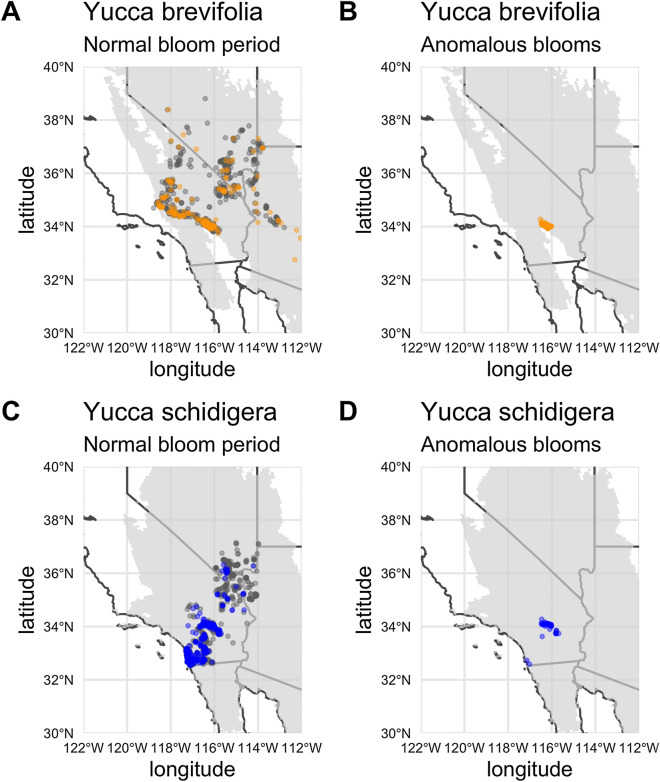
The spatial distribution of occurrence records used in the analyses (2009–2020). The gray polygon in the background of each plot portrays the presumed range for the species. Colored dots indicate flowering presences, and gray-scale dots indicate flowering absences. This figure was generated using R version 4.0.3^23^ and the R packages rnaturalearthhires (https://docs.ropensci.org/rnaturalearthhires/), ggplot2^24^, and spData (https://nowosad.github.io/spData/).

**Figure 2:**
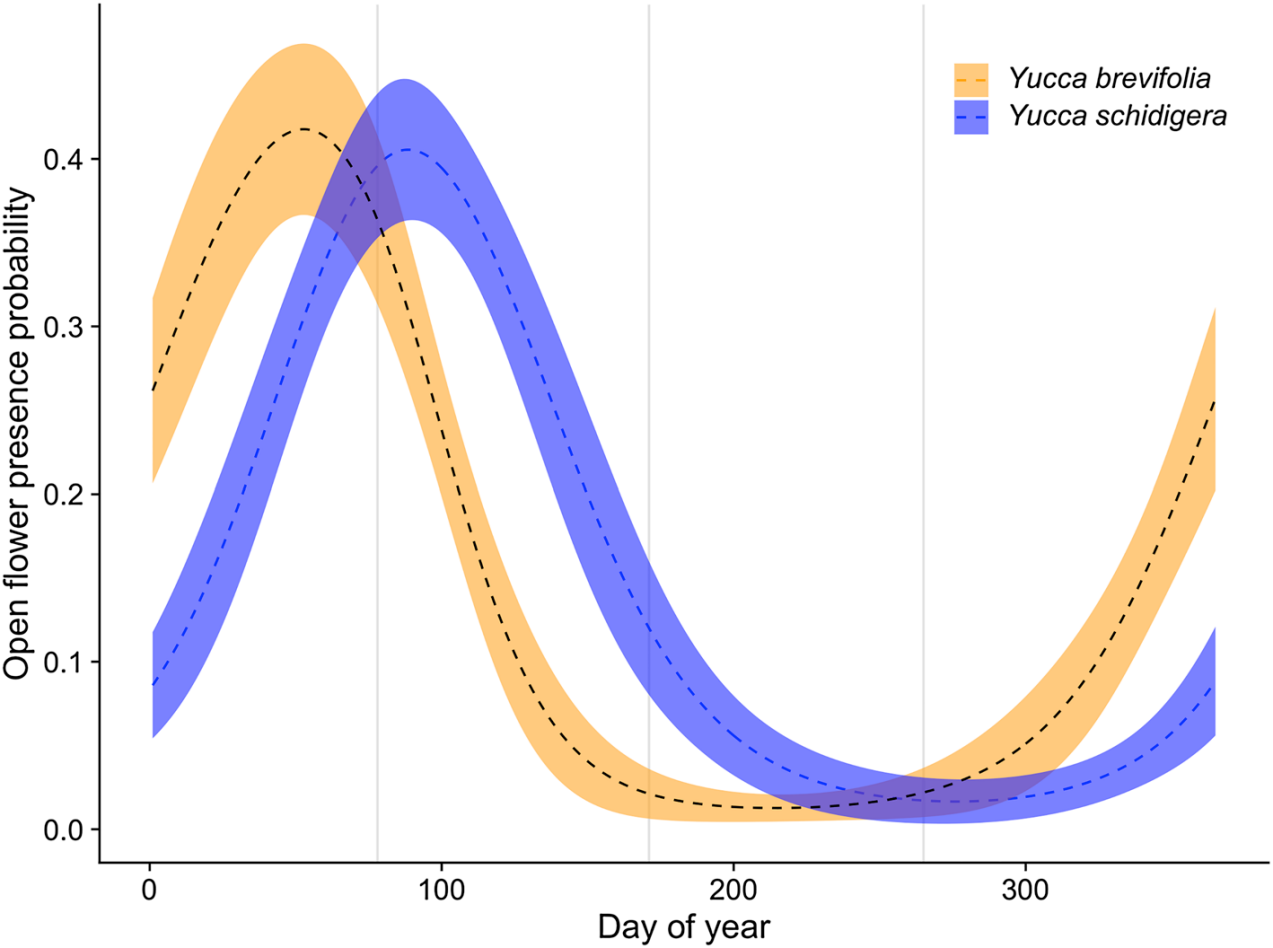
The probability of open flower presences given the day of year for the two species under normal bloom conditions.

We scored a total of 1375 presence/absence observation records for *Y. brevifolia* and 1637 for *Y. schidigera* ranging from 2009 to 2020. Figure 1 shows the spatial distribution of normal and anomalous flowering for both species. Observations of the anomalous flowering were mostly spatially localized to the Joshua Tree National Park area. We used a cyclical GAM to determine the probability of flowering of both species over the course of the year, summarized in Fig. 2. *Y. brevifolia* flowers earlier in a typical year than *Y. schidigera*, with probability of flowering peaking in late February into early March before effectively ending in late April. In contrast, open flowers peak in *Y. schidigera* in late March into early April with full cessation by the end of June. For both species, flowering probability peaks at just above 40%, which may be an overestimate given biases towards photographs of *Yucca* in flower^1^.

### Model calibration, model testing, and final model fitting

We used general linear models with GDDs, accumulated precipitation, and daylength as covariates, all previously hypothesized to determine *Yucca* blooming in desert environments. We fit 10 replicates with different training and testing subsets for both species and accumulation periods. For *Y. brevifolia* using 30-day accumulations, the best model based on AICc was most commonly (nine out of 10 times) one that had a three-way interaction between GDD, accumulated precipitation and second-order polynomial daylength term. Across the ten replicates, we found highly consistent and performant AUC scores for training and testing (average AUC_train_: 0.90733, average AUC_test_: 0.89714). We found similar results for the 120-day accumulation model for *Y. brevifolia*; all top models had a three-way interaction and second-order polynomial daylength term, with marginally better model validation statistics compared to the 30-day models for both training and testing (average AUC_train_: 0.91379; average AUC_test_: 0.90315). Mojave yucca (*Y. schidigera*) model results were similar, with the 120-day climate accumulation performing models slightly better than the 30-day models (AUC_train_: 0.88326, AUC_test_: 0.85829 for 30-day; AUC_train_: 0.89528, AUC_test_: 0.87614 for 120-day). The most common best model (six out of 10 times) for *Yucca schidigera* using the 120-day climate accumulations also included a three-way interaction among climate variables and a second-order polynomial term for daylength. Based on these results, we opted to fit final models using the full dataset (all data from normal blooming periods) for the 120-day climate accumulations, given slightly superior performance based on AUC statistics. After another model selection step during model fitting, the final model for both species also included a three-way interaction and a non-linear response to daylength, with results summarized in Table 1.

**Table 1:** Final best-fit models for each species.

Term	*Y. brevifolia*	*Y. schidigera*
N	1084	1378
Intercept	− 4.28 ± 0.48**	− 2.46 ± 0.25**
GDD	− 3.25 ± 0.55**	− 0.66 ± 0.27*
Average precipitation	0.19 ± 0.49	1.48 ± 0.20**
Poly1 (daylength)	− 4.10 ± 15.4	59.3 ± 11.4**
Poly2 (daylength)	− 73.7 ± 20.5**	− 70.9 ± 10.8**
GDD: average precipitation	− 0.04 ± 0.61	1.75 ± 0.30**
GDD:poly1 (daylength)	− 3.60 ± 17.7	19.0 ± 11.3
GDD:poly2 (daylength)	− 32.3 ± 23.3	3.32 ± 12.5
Average precipitation:poly1 (daylength)	− 25.3 ± 16.5	− 13.8 ± 9.34
Average precipitation:poly2 (daylength)	− 81.0 ± 23.6**	5.25 ± 11.5
GDD: average precipitation: poly1 (daylength)	− 44.2 ± 21.5*	− 63.1 ± 12.3**
GDD: average precipitation: poly2 (daylength)	− 63.5 ± 28.8*	44.7 ± 16.3**
ΔAIC	16.6	4.48
R^2^	0.41	0.39

Occurrence records with incomplete climate data were filtered from the datasets used to fit these models. The best model includes a three-way interaction between growing degree days, precipitation, and photoperiod. A star is used to denote p < 0.05 and a double star indicates p < 0.01.

**Figure 3:**
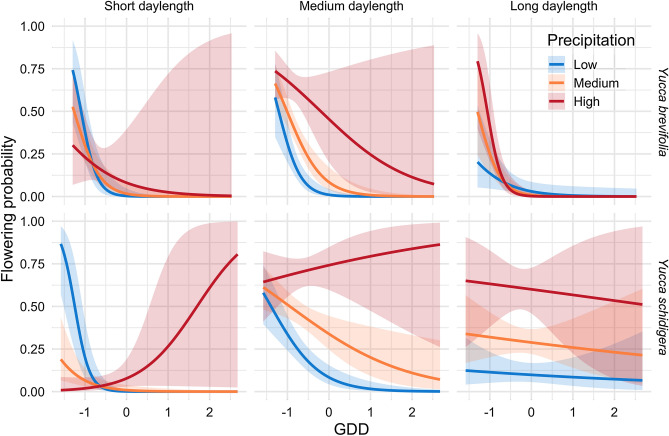
Predicted probability of flowering given scaled daylength, scaled GDD, and scaled average precipitation conditions for *Yucca brevifolia* (top panel) and *Yucca schidigera* (bottom panel).

The effects plots shown in Fig. 3 show the interrelationships found in our final 120-day climate accumulation models. We found that these two *Yucca* species bloom when it is cooler, even if it is dry. Later in the season, high temperatures tend to decrease the probability of flowering unless conditions are wet. For *Y. brevifolia*, medium daylength, low temperatures, and high precipitation increase the probability of blooming. For *Y. schidigera*, high precipitation and higher temperatures increase the probability of blooming if daylengths are short. *Y. schidigera*, which blooms later (Fig. 2) is more likely to bloom in longer daylengths, unlike *Y. brevifolia*, which only blooms late-season if cold and wet.

### Anomalous bloom prediction

Using the final 120-day climate accumulation models for both species, we generated prediction probabilities for observed presences and absences from the 2018 to 2019 winter season anomalous bloom. These probabilities were converted into predicted presences (1) and absences (0) using MaxKappa, LPT, and LPT 5% values as thresholds, and then the accuracy of these predictions were assessed (Table 2). For both species, we found higher commission (false positive) error rates than omission (false negative) error rates. The LPT threshold generated the highest commission error rates and the MaxKappa and LPT 5% thresholds were roughly equivalent in their commission error rates for the anomalous bloom season. We found generally low omission rates across all thresholds.

**Table 2:** Anomalous bloom prediction test results.

Species	N	Overall accuracy	Commission error (false positive)	Omission error (false negative)
*Y. brevifolia*	252	MaxKappa: 61%	MaxKappa: 32.1%	MaxKappa: 6.7%
		LPT: 48%	LPT: 51.9%	LPT: 6.7%
		LPT 5%: 57.5%	LPT 5%: 37.7%	LPT 5%: 4.7%
*Y. schidigera*	211	MaxKappa: 68%	MaxKappa: 27%	MaxKappa: 4.7%
		LPT: 49.7%	LPT: 50.2%	LPT: 4.7%
		LPT 5%: 68%	LPT 5%: 26.5%	LPT 5%: 5.2%

## Discussion

We assembled a unique dataset of range-wide observations of *Yucca* available from iNaturalist. These provided the bases for determining presence and absence of flowers for our two focal species, and while flowering *Yucca* are likely to be more photographed, we still had sufficient absences for these common and iconic species to use in downstream models. These models were effective at predicting flowering phenology of the two focal species with generally high accuracy during the normal flowering season. Unlike other frameworks for predicting phenology, our approach is not to estimate an onset, median or termination timing of a phenophase. Rather, our goal was to determine whether climate and daylength covariates provide a basis for predicting the probability of open flowers during normal and anomalous bloom periods. This approach is enabled by having dense reporting of presence-absence data generated by growing community science resources.

Model fitting revealed that probability of flowering is determined by complex interactions between climate and daylength, suggesting the critical importance of climate context. This importance for arid-adapted *Yucca* is in line with other studies of phenology of desert plants, from Saguaro in the desert Southwest of North America^9^ to lilies in arid environments in Africa^25^. Yet, we were surprised that flowering of *Yucca* does not necessarily always rely on increased precipitation. We expected precipitation would be a critical limiting factor in desert environments, and for Mojave yucca in particular, precipitation is a strong driver positively influencing the odds of flowering during spring and summer. However, for both species, flowering odds are also relatively high during cold and drier conditions earlier in the season (Fig. 3). These results may relate to precipitation falling as snow rather than rain during late winter—another avenue for further exploration in follow up studies.

Joshua trees and Mojave yucca have different growth forms and are of vastly different sizes at maturity, and therefore, may be expected to react differently to climatic drivers. However, our findings indicate that the same interacting climate variables drive flowering phenology for both species, and the overall shape of their seasonal phenology curves are similar (Fig. 2). The main differences in our models are likely attributed to adapted differences in overall bloom timing; in particular, *Y. schidigera* generally blooms later in the year than *Y. brevifolia* (Fig. 2). For example, *Y. brevifolia* only blooms under highest daylength conditions if it is unusually cold and wet, while *Y. schidigera* flowering odds can often exceed 50% under warm and wet conditions when daylength is long (Fig. 3). Conversely, while *Y. brevifolia* has better odds of blooming in cold, dry conditions early in the year, even under wet conditions it can bloom with odds above 25%. In contrast, *Y. schidigera* is rarely in bloom in colder, wetter early season conditions.

We also note the importance of including a polynomial term for daylength (Table 1), which always dramatically improved models (Table 1). The outcome, clearly visible in Fig. 3, is that responses of phenology are strongly non-linear across gradients. In sum, our work corroborates the importance of context dependence, finding that daylength, temperature, and precipitation interact in complex, nonlinear ways to influence flowering times.

A key question we sought to answer in this work is whether we predict anomalous flowering events. Accelerating climate change means that species will experience conditions outside the range experienced for centuries. How species respond phenologically to these novel conditions is an area of active research^26^, but the focus has predominantly been on using yearly anomaly data, e.g. warmest years on record or via warming experiments^27^. Our efforts here are trying to predict a seasonal anomaly, where plants seasonally flowered outside of their presumed normal periods (e.g. in fall rather than spring). A key question of interest was whether the fall-winter bloom in 2018–2019 was itself triggered by anomalous climate conditions mirroring those of the usual bloom period.

We examined this question by testing whether models, which were fit using data from years with a known normal blooming period, were able to predict presences and absences during the 2018–2019 fall-winter season. Our results show that we can predict absences with low error rates (4.7–6.7%, Table 2). However, these models had much higher rates for false positives (32.1–50.2%, Table 2). Our model predicts more anomalous blooming than actually observed. This suggests that, while *Yucca* might have been triggered to bloom by atypical cooler and wetter conditions, there are still factors not included in our models that limited the extent of anomalous blooming.

It remains possible that co-evolution between *Yucca* and their obligate pollinator or florivore community^28^ may extend to how phenology is cued. It may also be that *Yucca* are responding not only to instantaneous climate conditions, such as mean photoperiod, but whether days are shortening or lengthening, and if so, it may be that the modeling approach used here is not sensitive enough to capture these types of more dynamic seasonal cues. Our work may also point to out-of-normal season blooming simply being more common and widespread than previously suspected, given broadly suitable climate conditions. A next step is to use growing community-science reporting of *Yucca* plants in flower to determine the rate of seasonally anomalous flowering from dense, range-wide community science observations enabled via resources such as iNaturalist.

Finally, we note the value of examining predictive power of models using climate measurements over shorter and longer temporal windows. While these different climate accumulation windows are by nature highly autocorrelated, we found that data from the longer temporal window led to modest improvement of models based on AUC statistics. It is likely that the longer temporal window captures more information about GDD and overall water input in the environment. For example, a classic paper by Beatley^5^ that focused on shrub phenology in the Mojave showed fall and winter rains were precursor triggers of phenological events in spring. We also note congruence with the findings of Clair and Hoines^13^, who showed strong positive correlations with the 30-year averages of temperature and precipitation and fruit and seed mass in Joshua trees, although they focused on broader temporal scale questions. Connecting these longer timescale and broad spatial phenology studies, and aligning lags over different time-scales is a frontier area in phenology research. Finally, our findings should not necessarily be extrapolated more broadly for arid-adapted plants, and traits such as perenniality or woody versus herbaceous habit with associated differences in costs for growth and reproduction, may condition thresholds for needed accumulation of heat or water.

We close by noting that phenology modeling is often treated as a one-off exercise where models are built, and results shared. We argue that the accelerating growth of new data resources and flexible modeling frameworks provide a means for models to iteratively improve. One key step towards this goal is faster annotation of phenology state. Here we hand-coded two key states in *Yucca* photographs^1^. These carefully vetted classifications can now provide the basis for more automated approaches for annotating photographs, e.g. via machine learning^29^. These new results can be fed into current models to test and improve model performance.

Expanding data resources for modeling flower presence is one key step, but the development of improved phenology models that include more fitness-relevant responses is also important, such as number of flowers or fruits, potentially in relation to vegetative biomass. Individual yucca plants, for example, do not bloom annually even in favorable conditions, because vegetative growth must precede production of a heavy, high-cost inflorescence^10^. More sophisticated species-level models that link the full range of environmental conditions populations experience across their range with seasonal vegetative and reproductive biomass proxies are uncommon, mostly due to data limitations. Rather, studies typically focus on single, local areas or transect approaches across broad-scales^30^, with associated limitations for further prediction or forecasting.

We argue that the ability to develop ecophysiological-guided, range-wide models are in reach, using the same community science photographs that so far have only been used to generate simple states such as open flower presence. Such models hold promise in helping to provide a basis for improved understanding of mechanisms underlying flowering, and better detection of anomalous blooming events and their consequences. For example, we don’t yet know if anomalous blooms produce fewer or greater flower numbers, as compared to normal periods. Do these blooms ultimately lead to the production of fruit and, if so, how much? Such next-step approaches are particularly critical and necessary, because as we experience more unusual weather phenomena and novel conditions, phenology prediction and understanding the consequences of phenological changes becomes even more challenging. As weather forecasting was improved by assimilating more data and building better process parameters, our hope is that similar methods with richer data types can improve the most difficult phenology prediction challenges.

## Methods

### Observation record accumulation

We chose to focus on *Y. brevifolia* and *Y. schidigera*, because original exploratory analyses of the *Yucca* dataset showed that some populations of these species were in bloom at abnormal times in the winter of 2018–2019. We used iNaturalist records for these two species, following the scoring methodology developed by Barve et al.^1^. We downloaded photographs from research-grade observations on iNaturalist for both species. Volunteers and authors used the software package ImageAnt (https://gitlab.com/stuckyb/imageant) to annotate if flowers (open or closed) and/or open flowers were present in the images. We note that there is debate regarding Joshua tree taxonomic delimitation with some considering eastern and western forms to be different species (*Y. brevifolia* and *Y. jageriana*) or subspecies. For the purposes of this work, we have kept these lumped. Future effort may be warranted to examine any subspecies or species-specific phenology differences.

A key annotation component was whether the whole plant or only part of a plant was visible in the image, which is essential for scores of “flowers absent”. If an image covers part of a plant and has no flower, it is possible that the plant either actually does not have flowers or may have flowers on its other parts not captured in the image. In cases where a scorer could not determine whole plant status from a photograph, we utilized an “uncertain” category. Full details of scoring best practices, which were followed here, is available in Barve et al.^1^. These best practices included having at least two independent volunteers annotate each image. In cases of conflict, annotations were checked by L.B., V.B., and R.P.G. and a final decision was made. Initial work from Barve et al.^1^ had scored plants observed on iNaturalist from 2009 through spring of 2019. The co-authors (L.B., V.B., and R.P.G.) score more records for *Y. brevifolia* and *Y. schidigera* to expand the temporal coverage through February 2020.

### Climate data collection

We used PRISM daily data, including both stabilized “recent years” layers, as well as the provisional “previous 6 month” data. For each *Yucca* record, we used the date and location to extract accumulated measures of growing degree days (GDD) and overall precipitation. Growing degree days and average precipitation were calculated for two different time spans: 30 days and 120 days before the observation. For GDD, we used a base temperature of 5 ℃ and no upper limit^11^. In any cases where GDD values were less than zero, we assigned a value of zero. Finally, we assembled daylength (or photoperiod) for each record based on location and date of observation using the “daylength” function in the R package geosphere^31^.

### Modeling framework

To test if flowering phenology models of *Y. schidigera* and *Y. brevifolia* performed better when using a shorter or longer temporal window from the date of observation, we assembled a total of four datasets—two per species with climate data from 30 days prior to the date of observation and 120 days prior to observation. Before model fitting, initial phenology annotations were processed to determine a final scoring of confirmed absences and presences of flowers based on the whole plant annotation of an image. Confirmed absences required that a whole plant was visible in the image and lacked flowers. Cases where only a part of a plant was visible and flowers were absent were not used further in analyses. We coded both flower presence and open-flower presence, which provide a means to infer unopened flowers (cases where a flower is present but open flowers are not). However, cases of confirmed unopened flowers were uncommon, and we therefore focus on the broad category of flower presence/absence rather than sorting into narrower phenophases. We also eliminated records that did not have values for all climate variables used for modeling. This led to a final set of 1336 records available for model fitting for *Y. brevifolia* and 1589 records for *Y. schidigera*. This total included both normal and anomalous flowering scoring, which was further subset below and used as the response variable in generalized additive models (GAMs) and generalized linear models (GLMs). All analyses were performed using R version 4.0.3^23^.

To visualize the general shape of seasonal flowering phenology curves, we fit a GAM for both species separately where the response variable was flower presence or absence and the predictor was ordinal day of the year using the mgcv and mgcViz R packages^32–34^. We used a logistic function (family = binomial()) and a cubic cyclic regression spline smoother. This smoother constrained the start and end of each phenology curve to match in value and first derivative, which is appropriate for cyclic biological phenomena^35^. GAM fitting showed expected flowering during late winter-late spring during cooler and wetter periods. We tested if indeed higher precipitation, cooler temperatures and shorter daylength interact to predict normal flowering, and if these predictors also successfully predict anomalous blooming. Our approach is as follows. First, we separated the anomalous bloom period of fall 2018 to winter 2019 from the complete datasets. Anomaly data was not used to train or validate the models. The remaining data were randomly split with 75% of records utilized for model training and 25% for model testing/validation. We then used a repeated cross-validation approach, randomly splitting our data into testing and training datasets ten times. This process was repeated for all four datasets (two species × two climate accumulation periods) independently, and these training datasets were used to calibrate models, discussed below, for each species with the 30-day and 120-day climate data. We used the glm() function with a binomial logit link function to test the ability of *GDD*, *average_precip* (average precipitation), and *daylength* to predict the probability of *flowers* for *Y. schidigera* and *Y. brevifolia*. All predictor variables were scaled to have mean zero and standard deviation of one before model fitting.

There is no reason to expect purely linear responses of flowering to climate drivers, and one predictor may interact with another. Therefore, we explored a suite of models that included second order polynomials for each predictor, as well as interactions. These more complex models often lead to high variance inflation, so we checked for such issues using the vif() function in R and did not consider models with predictors where variance inflation factors or VIFs > 5. We thus examined multiple models, ranging from purely linear effects with no interactions to more complicated ones with more interactions and polynomial terms. We used model selection approaches to determine the best models for those that passed screening via the Akaike’s information criteria (AIC) and report the ΔAIC and Akaike weights for that model (see below).

### Model validation and ability of models to predict anomalous blooming

After calibrating models, we tested their performance on the withheld test data. To evaluate model performance for both training and test data, we used the R package ROCR^36^ to calculate area under the curve (AUC) scores. We considered models to be performant in cases where AUC scores were high (> 0.8), and where calibrated models show ability to generalize. We evaluated ability to generalize by determining if test AUCs were lower than model calibration AUCs. Cases where training and testing AUC were both high were considered to be best-case scenarios. Metrics were calculated for all repeated cross-validations.

Finally, we examined model validation statistics for 30 and 120-day climate accumulation models across all repeated cross validations for each species, with the goal of determining if one or the other climate accumulation dataset was more predictive. While both climate accumulation models were performant, the 120-day models consistently outperformed the 30-day ones for both species (see “Results” below). We thus used all the data for both species to fit final models for 120-day climate accumulations and selected the best models based on AIC.

Using the predict() function in R, we used these final models to calculate the probability of flowering for records collected during the anomalous bloom period. We used thresholds based on model calibrations, including the maximum kappa (MaxKappa), least presence threshold (LPT), and 5% least presence threshold (5% LPT), to convert those probabilities into predicted presence or absence. These are commonly applied thresholds in the literature^37^, with MaxKappa representing the maximum of observed versus expected accuracy, and LPT thresholds representing minimum values where all or 95% of the plants in flower are correctly classified as such. Then, we determined the true and false positive and negative rates for the model predictions compared to the actual known flowering state of the records collected during the anomaly. If unusual fall climate conditions mimicked spring conditions known to drive flowering, we expected calibrated models to be useful for predicting anomalous flowering.

## Data Availability

All data and code used for analyses in this paper have been made available in Dryad (10.5061/dryad.02v6wwq49). Most of the phenology scoring used here were deposited into the plantphenology.org portal as part of efforts included in Barve et al., 2020. New scoring that was not part of the previous deposition will also be deposited to assure the broadest, and most integrated reuse. They are also available in Dryad. Finally, the original set of records annotated here are citable at the following 10.15468/dl.xxxwpx^36^.
